# Supplementary material for: Genome-centric analyses of 165 metagenomes show that mobile genetic elements are crucial for the transmission of antimicrobial resistance genes to pathogens in activated sludge and wastewater
Source: Microbiol Spectr. 2024 Jan 30;12(3):e02918-23. doi: 10.1128/spectrum.02918-23 (PMC10913551; doi:10.1128/spectrum.02918-23)
Supplement: Supplemental figures — Fig. S1 to Fig. S7. [file spectrum.02918-23-s0001.pdf]

Genome-centric analyses of 165 metagenomes show that mobile genetic elements are crucial for the transmission of antimicrobial resistance genes to pathogens in activated sludge and wastewater

Nafi'u Abdulkadir, Joao Pedro Saraiva, Junya Zhang, Hauke Harms, Stefan Stolte, Osnat Gillor, Hauke Harms and Ulisses Nunes da Rocha

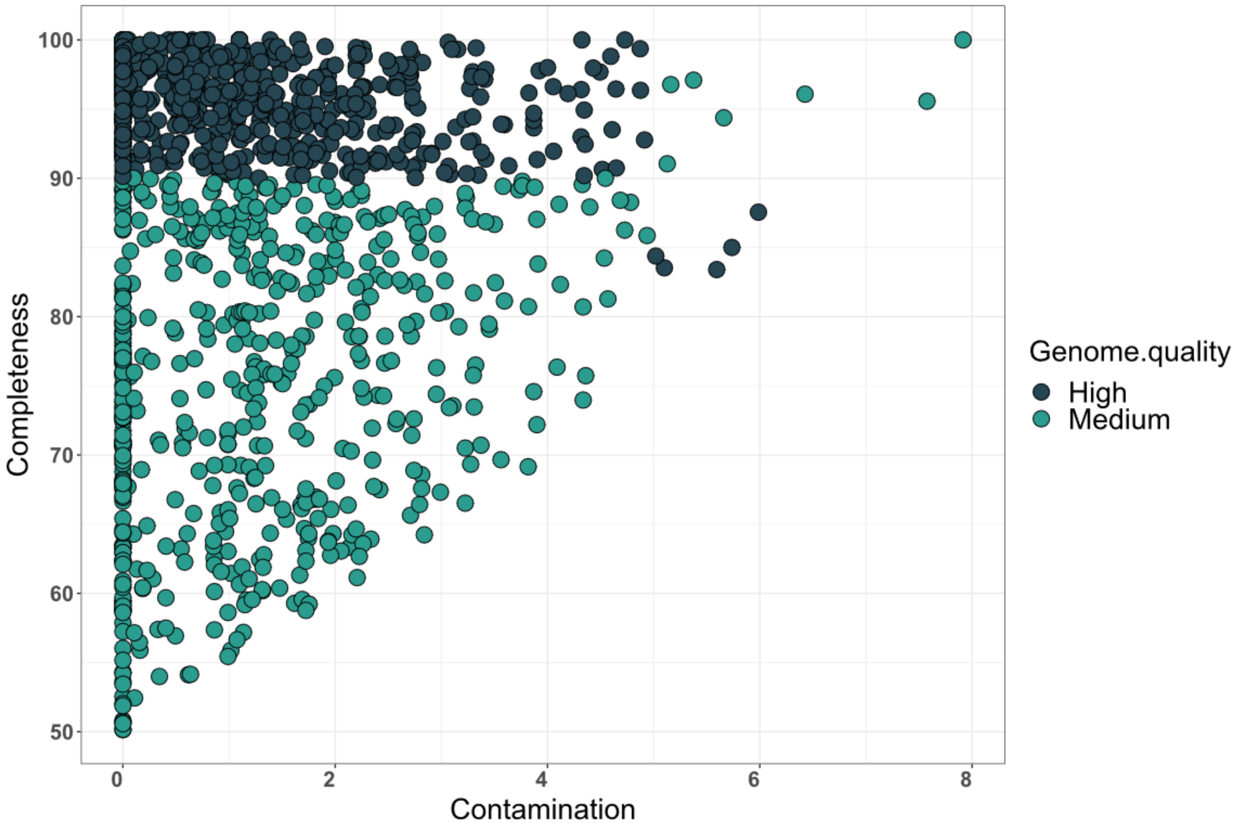

FIG S1. Genome quality of genome operational taxonomic units**.** A scatter plot showing the contamination and completeness levels of the 1,204 genome operational taxonomic units (gOTUs). Each point is coloured according to its quality score. The quality score is calculated the completeness minus five times contamination (% Completeness – (5 x % contamination). Medium-quality MAGs have completeness higher than 50% and contamination lower than 10%. High-quality MAGs have completeness higher than 90% and contamination lower than 5%. All MAGs have a quality score higher than or equal to 50.

Genome-centric analyses of 165 metagenomes show that mobile genetic elements are crucial for the transmission of antimicrobial resistance genes to pathogens in activated sludge and wastewater

Nafi'u Abdulkadir, Joao Pedro Saraiva, Junya Zhang, Hauke Harms, Stefan Stolte, Osnat Gillor, Hauke Harms and Ulisses Nunes da Rocha

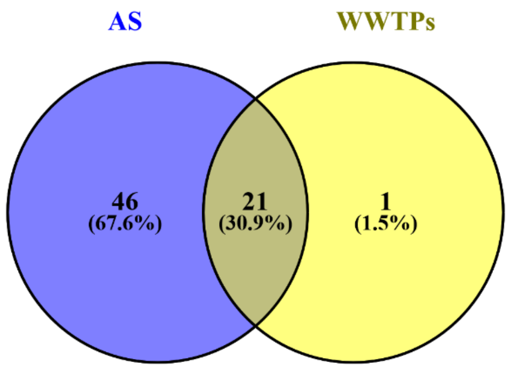

FIG S2. A Venn diagram shows the number and percentage of phyla shared between the activated sludge and wastewater samples.

Genome-centric analyses of 165 metagenomes show that mobile genetic elements are crucial for the transmission of antimicrobial resistance genes to pathogens in activated sludge and wastewater

Nafi'u Abdulkadir, Joao Pedro Saraiva, Junya Zhang, Hauke Harms, Stefan Stolte, Osnat Gillor, Hauke Harms and Ulisses Nunes da Rocha

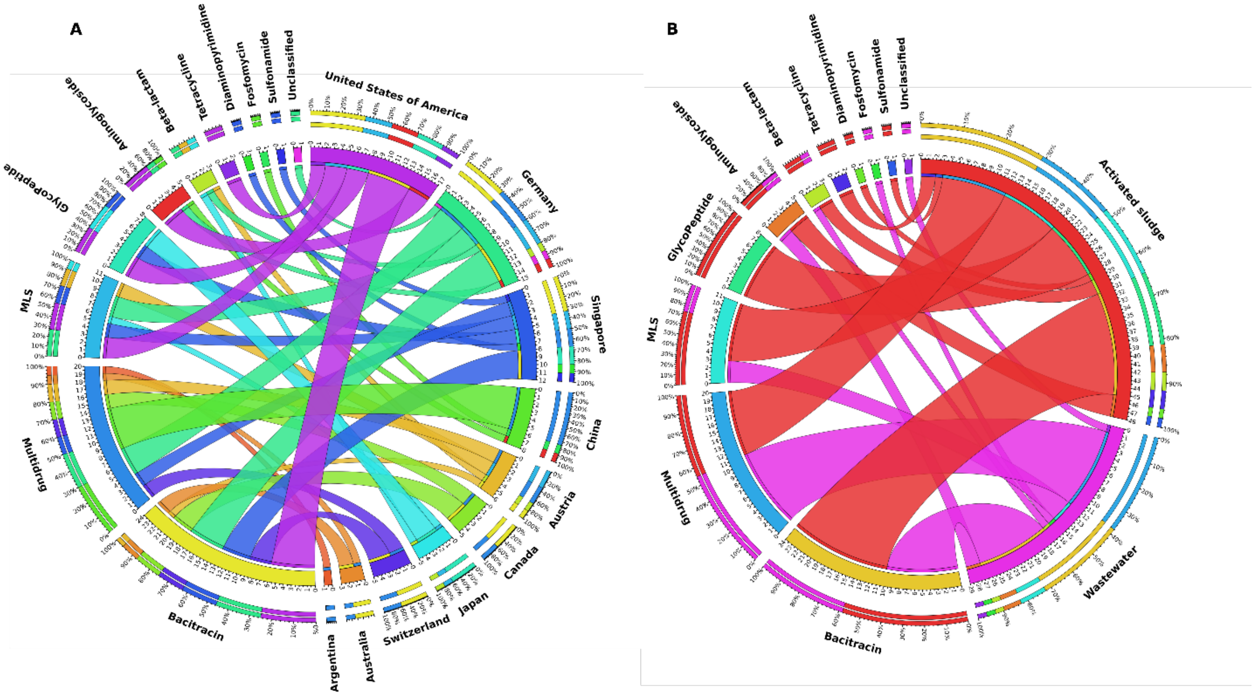

FIG S3. The abundance of antibiotic resistance gene classes in identified different sources and locations. (A) Circos showing the abundance of antibiotic resistance gene classes in different sample locations. The samples were retrieved from 14 different locations covering five continents. (B) Circos showing the abundance of antibiotic resistance gene classes in activated sludge and wastewater metagenomes.

Genome-centric analyses of 165 metagenomes show that mobile genetic elements are crucial for the transmission of antimicrobial resistance genes to pathogens in activated sludge and wastewater

Nafi'u Abdulkadir, Joao Pedro Saraiva, Junya Zhang, Hauke Harms, Stefan Stolte, Osnat Gillor, Hauke Harms and Ulisses Nunes da Rocha

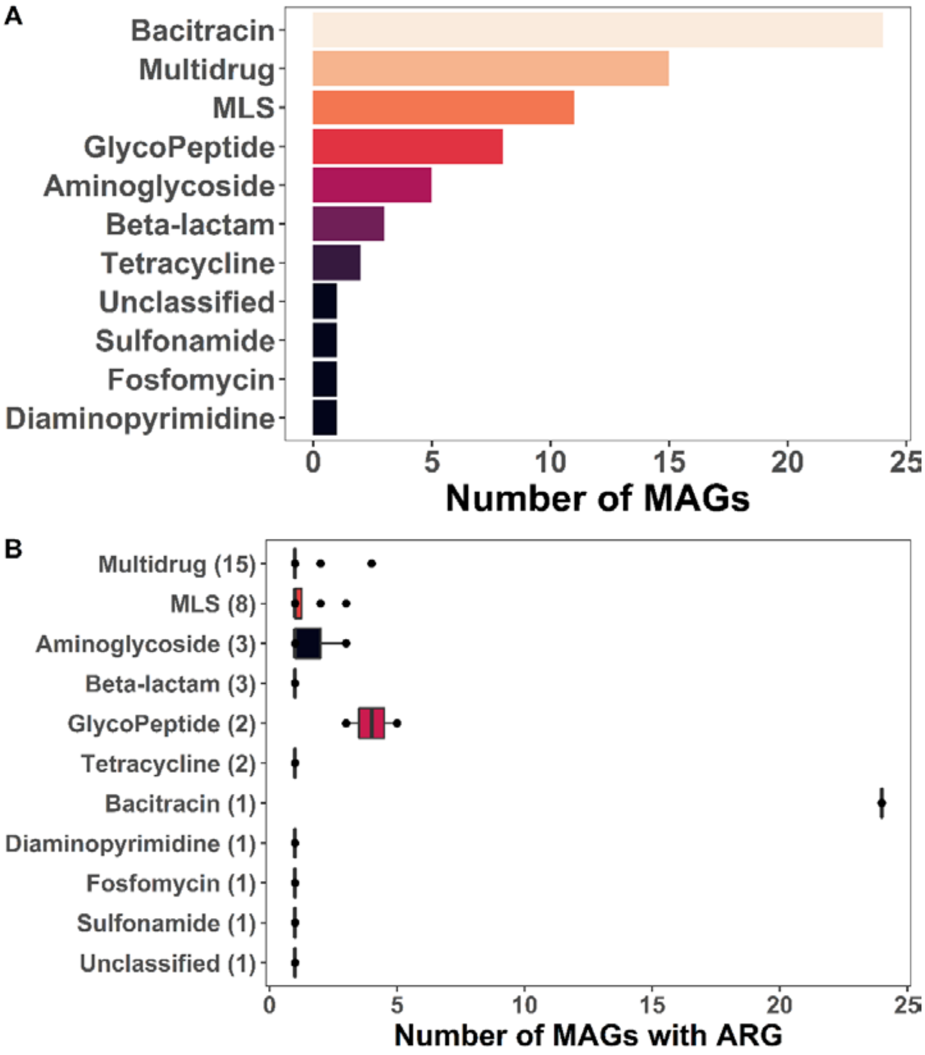

FIG S4. Number of metagenome-assembled genomes (MAGs) with resistance genes. (A) A bar plot shows the number of MAGs containing antibiotic resistance genes (ARGs) belonging to each class. (B) Boxplots showing the number of MAGs containing each ARG within the ARG classes present in the ubiquitous, widespread, and common categories from Fig. 2b. The parentheses are the number of ARGs within the ARG class.

Genome-centric analyses of 165 metagenomes show that mobile genetic elements are crucial for the transmission of antimicrobial resistance genes to pathogens in activated sludge and wastewater

Nafi'u Abdulkadir, Joao Pedro Saraiva, Junya Zhang, Hauke Harms, Stefan Stolte, Osnat Gillor, Hauke Harms and Ulisses Nunes da Rocha

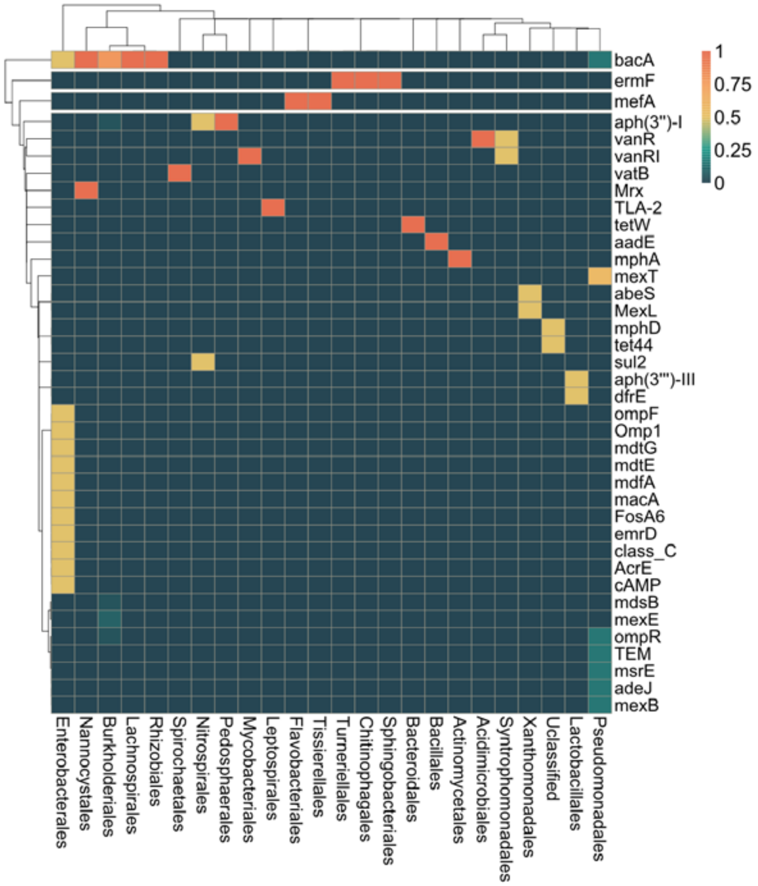

FIG S5. Prevalence of antimicrobial resistance gene (AMR) classes in activated sludge (AS) and wastewater (WW) bacterial communities**.** A heatmap shows AMR gene classes' prevalence in the AS and WW at the order level. The dendrogram is based on hierarchical clustering with Ward distance between the ARG class prevalence among the order.

Genome-centric analyses of 165 metagenomes show that mobile genetic elements are crucial for the transmission of antimicrobial resistance genes to pathogens in activated sludge and wastewater

Nafi'u Abdulkadir, Joao Pedro Saraiva, Junya Zhang, Hauke Harms, Stefan Stolte, Osnat Gillor, Hauke Harms and Ulisses Nunes da Rocha

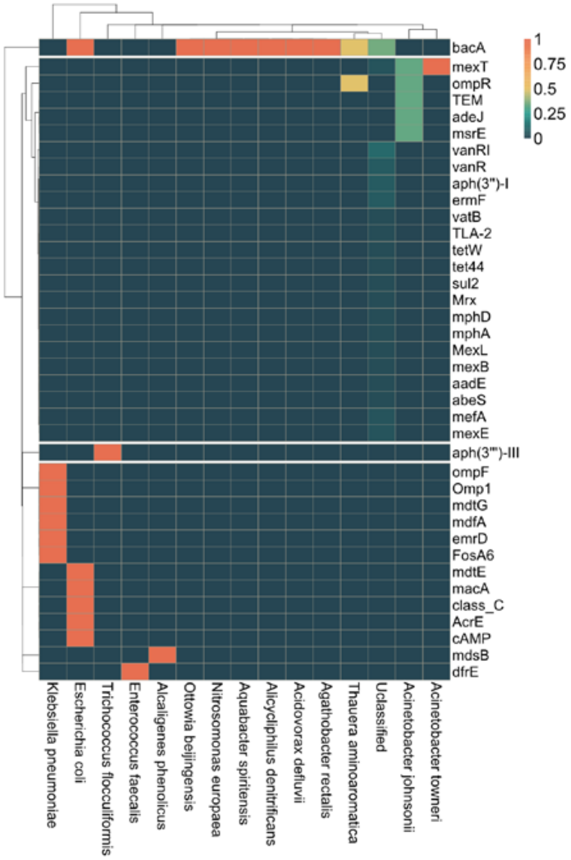

FIG S6. Prevalence of antimicrobial resistance gene (AMR) classes in activated sludge (AS) and wastewater (WW) bacterial species. A heatmap showing the prevalence of AMR gene classes in the AS and WW at species level. The dendrogram is based on hierarchical clustering with Ward distance between the ARG class prevalence among the species.

Genome-centric analyses of 165 metagenomes show that mobile genetic elements are crucial for the transmission of antimicrobial resistance genes to pathogens in activated sludge and wastewater

Nafi'u Abdulkadir, Joao Pedro Saraiva, Junya Zhang, Hauke Harms, Stefan Stolte, Osnat Gillor, Hauke Harms and Ulisses Nunes da Rocha

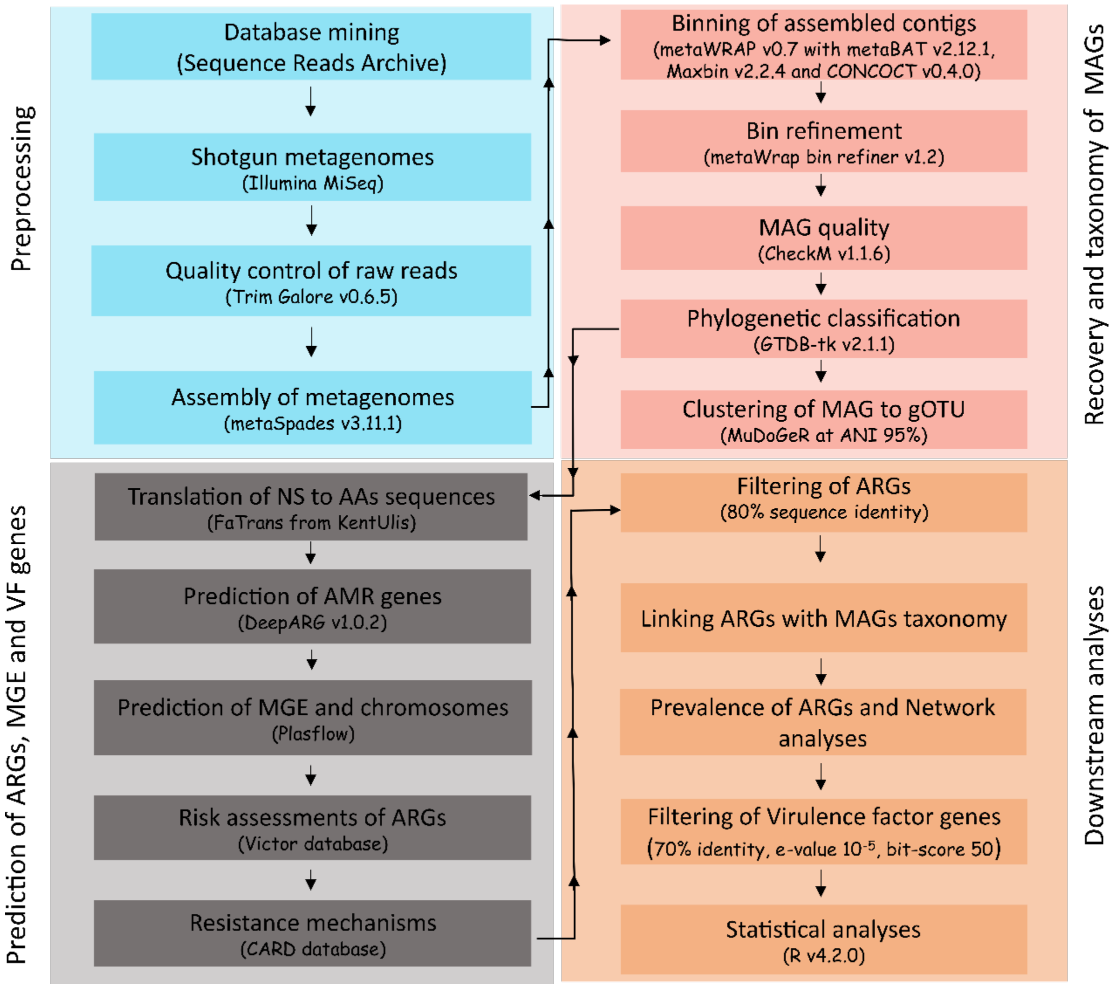

FIG S7. Study workflow showing a details overview of the tools/devices used. The study was conducted by selecting 165 publicly available metagenomes from sequence reads Achieves (SRA) belonging to wastewater and activated sludge from Terrestrial Metagenome Database (TMDB) (1). The metagenomes were assembled with metaSPAdes (2), and genome recovery was performed using MetaWRAP (3). Taxonomic classification was determined using the genome taxonomy database (GTDB-tk) (4). DeepARG (5) was used to predict antibiotic resistance genes (ARGs). Plasmids and chromosomes were predicted using the Plasflow model (6). The MAGs were dereplicated using SpecDiff implemented in MuDoGeR (7), and Gephi (8) was used for co-occurrence network analyses. All the downstream analyses were performed in R studio.

REFERENCES

1. Corrêa FB, Saraiva JP, Stadler PF, da Rocha UN. 2020. TerrestrialMetagenomeDB: a public repository of curated and standardized metadata for terrestrial metagenomes. Nucleic Acids Res 48:D626–D632.

2. Nurk S, Meleshko D, Korobeynikov A, Pevzner PA. 2017. metaSPAdes: a new versatile metagenomic assembler. Genome Res 27:824–834.

3. Uritskiy GV, DiRuggiero J, Taylor J. 2018. MetaWRAP—a flexible pipeline for genome-resolved metagenomic data analysis. Microbiome 6:158.

4. Chaumeil P-A, Mussig AJ, Hugenholtz P, Parks DH. 2020. GTDB-Tk: a toolkit to classify genomes with the Genome Taxonomy Database. Bioinformatics 36:1925–1927.

5. Arango-Argoty G, Garner E, Pruden A, Heath LS, Vikesland P, Zhang L. 2018. DeepARG: a deep learning approach for predicting antibiotic resistance genes from metagenomic data. Microbiome 6:23.

6. Krawczyk PS, Lipinski L, Dziembowski A. 2018. PlasFlow: predicting plasmid sequences in metagenomic data using genome signatures. Nucleic Acids Res 46:e35.

7. MuDoGeR - Google Search. https://www.google.com/search?client=firefox-b-e&q=MuDoGeR. Retrieved 1 August 2022.

8. Bastian M, Heymann S, Jacomy M. 2009. Gephi: An Open Source Software for Exploring and Manipulating Networks. 1. Proc Int AAAI Conf Web Soc Media 3:361–362.
