# Supplementary material for: Genome-centric analyses of 165 metagenomes show that mobile genetic elements are crucial for the transmission of antimicrobial resistance genes to pathogens in activated sludge and wastewater
Source: Microbiol Spectr. 2024 Jan 30;12(3):e02918-23. doi: 10.1128/spectrum.02918-23 (PMC10913551; doi:10.1128/spectrum.02918-23)
Supplement: Supplemental text — Supplemental methods. [file spectrum.02918-23-s0002.pdf]

Genome-centric analyses of 165 metagenomes show that mobile genetic elements are crucial for the transmission of antimicrobial resistance genes to pathogens in activated sludge and wastewater

Nafi'u Abdulkadir, Joao Pedro Saraiva, Junya Zhang, Hauke Harms, Stefan Stolte, Osnat Gillor, Shai Arnon and Ulisses Nunes da Rocha

Supplementary methods

Metagenome quality control and assembly. The sequence data of each metagenome were processed for quality controls to remove short and low-quality reads and adapter contamination using Trim Galore v0.6.5 implemented in MetaWRAP (1) according to the developer's instructions. The trimmed reads were subjected to the *de-novo* assembly through metaSPades v3.11.1 using default parameters (2) implemented in metaWRAP v0.7 (1). The assembled contigs with lengths lower than 1,000 nt (bp) were excluded from further analysis as they might not provide resolution to the species level.

Reconstruction of prokaryotic metagenome-assembled genomes. The binning of assembled contigs was performed using the metaWRAP v0.7 (1) pipeline (a tool designed for multiple binning with metaBAT v2.12.1 (3), Maxbin v2.2.4 (4) and CONCOCT v0.4.0 (5) to generate prokaryotic metagenome-assembled genomes (MAGs). The initial evaluation of metagenomics bins were performed using the metaWRAP binning_refiner v1.2 (1). Bins were subjected to quality assessments based on three primary measures: quality score, completeness, and contamination. Completeness, contamination and stains heterogeneity were estimated using CheckM v1.1.6 (6) via the lineage-specific workflow, while the quality score was calculated as described by (7). The bins recovered by metaWrap were assigned as MAGs with a quality score above 50% using equation 1.

$$\textit{quality score} = \textit{completeness}\ (\%) - (5 \times \textit{contamination}\ (\%)) \qquad (1)$$

In Equation (1), completeness is defined as the genome quality based on the presence of single-copy marker genes, and contamination is the estimation of genome quality as indicated by the presence of multiple copies of marker genes (7)

Furthermore, we selected genome operational taxonomic units (gOTUs) as a proxy of species from our MAGs resulting in 1,204 gOTUs. We defined gOTUs as a cluster of MAGs with the same taxonomy at 0.95 average nucleotide identity distance (ANI). Next, we used Anisplitter to split the genome of the same taxonomy as described (8).

Phylogenetic analysis and taxonomic classification of MAGs. Taxonomic classification was determined for 5916 MAGs using Genome Taxonomy Database tool kits (GTDB-Tk v2.1.1) (9) for bacterial and archaeal genomes. GTDB-Tk relies on 120 bacterial and 122 archaeal marker genes (9)**.** Further, the taxonomic assignments of MAGs with resistance genes that GTDB-tk did not classify to at least genus levels were further classified using the Microbial Genome Atlas (MIGA) (10) to see if we have more pathogenic species within the ESKAPE (11) panel. The phylogenetic tree was built using FastTree2 (12) with concatenated protein sequences of MAGs with resistance genes above 80% amino acid sequence identity. Next, the tree was visualized using iTOL v5.5 (13).

REFERENCES

1. Uritskiy GV, DiRuggiero J, Taylor J. 2018. MetaWRAP—a flexible pipeline for genome-resolved metagenomic data analysis. Microbiome 6:158.

2. Nurk S, Meleshko D, Korobeynikov A, Pevzner PA. 2017. metaSPAdes: a new versatile metagenomic assembler. Genome Res 27:824–834.

3. Kang DD, Li F, Kirton E, Thomas A, Egan R, An H, Wang Z. 2019. MetaBAT 2: an adaptive binning algorithm for robust and efficient genome reconstruction from metagenome assemblies. PeerJ 7:e7359.

4. Wu Y-W, Simmons BA, Singer SW. 2016. MaxBin 2.0: an automated binning algorithm to recover genomes from multiple metagenomic datasets. Bioinformatics 32:605–607.

5. Alneberg J, Bjarnason BS, Bruijn I de, Schirmer M, Quick J, Ijaz UZ, Lahti L, Loman NJ, Andersson AF, Quince C. 2014. Binning metagenomic contigs by coverage and composition. 11. Nat Methods 11:1144–1146.

6. Parks DH, Imelfort M, Skennerton CT, Hugenholtz P, Tyson GW. 2015. CheckM: assessing the quality of microbial genomes recovered from isolates, single cells, and metagenomes. Genome Res 25:1043–1055.

7. Parks DH, Rinke C, Chuvochina M, Chaumeil P-A, Woodcroft BJ, Evans PN, Hugenholtz P, Tyson GW. 2017. Recovery of nearly 8,000 metagenome-assembled genomes substantially expands the tree of life. 11. Nat Microbiol 2:1533–1542.

8. Rocha UN da, Kasmanas JC, Kallies R, Saraiva JP, Toscan RB, Štefanič P, Bicalho MF, Correa FB, Baştürk MN, Fousekis E, Barbosa LMV, Plewka J, Probst A, Baldrian P, Stadler P, Consortium C-T. 2022. MuDoGeR: Multi-Domain Genome Recovery from metagenomes made easy. bioRxiv https://doi.org/10.1101/2022.06.21.496983.

9. Chaumeil P-A, Mussig AJ, Hugenholtz P, Parks DH. 2020. GTDB-Tk: a toolkit to classify genomes with the Genome Taxonomy Database. Bioinformatics 36:1925–1927.

10. Rodriguez-R LM, Gunturu S, Harvey WT, Rosselló-Mora R, Tiedje JM, Cole JR, Konstantinidis KT. 2018. The Microbial Genomes Atlas (MiGA) webserver: taxonomic and gene diversity analysis of Archaea and Bacteria at the whole genome level. Nucleic Acids Res 46:W282–W288.

11. Mulani MS, Kamble EE, Kumkar SN, Tawre MS, Pardesi KR. 2019. Emerging Strategies to Combat ESKAPE Pathogens in the Era of Antimicrobial Resistance: A Review. Front Microbiol 10:539.

12. Price MN, Dehal PS, Arkin AP. 2010. FastTree 2 – Approximately Maximum-Likelihood Trees for Large Alignments. PLOS ONE 5:e9490.

13. Letunic I, Bork P. 2019. Interactive Tree Of Life (iTOL) v4: recent updates and new developments. Nucleic Acids Res 47:W256–W259.
