# Supplementary material for: Genome-centric analyses of 165 metagenomes show that mobile genetic elements are crucial for the transmission of antimicrobial resistance genes to pathogens in activated sludge and wastewater
Source: Microbiol Spectr. 2024 Jan 30;12(3):e02918-23. doi: 10.1128/spectrum.02918-23 (PMC10913551; doi:10.1128/spectrum.02918-23)
Supplement: Table S2 — Summary of sequence read characteristics. [file spectrum.02918-23-s0004.pdf]

Table S2. Summary of the sequence reads characteristics including the number of reads and base pairs of metagenomes retrieved from activated sludge and wastewater.

	Activated sludge			Wastewater		
	Reads count (bp)	Base pairs (bp)	Library fragment (bp)	Reads count (bp)	Base pairs (bp)	Library fragment (bp)
Max	8.68×10^{7}	2.60×10^{10}	602	8.27×10^{7}	2.48×10^{10}	302
Min	1.82×10^{6}	6.91×10^{8}	150	3.79×10^{6}	8.79×10^{8}	153
Ave	3.12×10^{7}	8.98×10^{9}	289.96	1.62×10^{7}	4.32×10^{9}	254.07
SD	1.80×10^{7}	5.48×10^{7}	69.69	1.72×10^{7}	5.29×10^{9}	52.63
Total	4.3×10^{9}	1.24×10^{12}	40014	4.36×10^{8}	1.17×10^{11}	6860
